# The use of error-category mapping in pharmacokinetic model analysis of dynamic contrast-enhanced MRI data

**DOI:** 10.1016/j.mri.2014.10.010

**Published:** 2015-02

**Authors:** Andrew B. Gill, Gayathri Anandappa, Andrew J. Patterson, Andrew N. Priest, Martin J. Graves, Tobias Janowitz, Duncan I. Jodrell, Tim Eisen, David J. Lomas

**Affiliations:** a Department of Radiology, Box 218, University of Cambridge, Cambridge Biomedical Campus, Cambridge, UK; b Department of Medical Physics, Box 152, Cambridge University Hospitals, Cambridge Biomedical Campus, Cambridge, CB2 0QQ, UK; c Department of Oncology, University of Cambridge, Cambridge Biomedical Campus, Cambridge, UK; d Department of Radiology, Box 219, Cambridge University Hospitals, Cambridge Biomedical Campus, Cambridge, CB2 0QQ, UK; e Cancer Research UK Cambridge Institute, Li Ka Shing Centre, Robinson Way, Cambridge, CB2 0RE, UK

**Keywords:** PK, pharmacokinetic, DCE-MRI, dynamic contrast-enhanced magnetic resonance imaging, eTM, extended Tofts model, AIF, arterial input function, AIC, Akaike information criterion, mRCC, metastatic renal cell carcinoma, MFA, multiple flip angles, ROI, region of interest, DCE-MRI, pharamacokinetic modeling, error analysis, metastatic renal cell carcinoma, repeatability

## Abstract

This study introduces the use of ‘error-category mapping’ in the interpretation of pharmacokinetic (PK) model parameter results derived from dynamic contrast-enhanced (DCE-) MRI data.

Eleven patients with metastatic renal cell carcinoma were enrolled in a multiparametric study of the treatment effects of bevacizumab. For the purposes of the present analysis, DCE-MRI data from two identical pre-treatment examinations were analysed by application of the extended Tofts model (eTM), using in turn a model arterial input function (AIF), an individually-measured AIF and a sample-average AIF. PK model parameter maps were calculated. Errors in the signal-to-gadolinium concentration ([Gd]) conversion process and the model-fitting process itself were assigned to category codes on a voxel-by-voxel basis, thereby forming a colour-coded ‘error-category map’ for each imaged slice.

These maps were found to be repeatable between patient visits and showed that the eTM converged adequately in the majority of voxels in all the tumours studied. However, the maps also clearly indicated sub-regions of low Gd uptake and of non-convergence of the model in nearly all tumours. The non-physical condition *v*_e_ ≥ 1 was the most frequently indicated error category and appeared sensitive to the form of AIF used.

This simple method for visualisation of errors in DCE-MRI could be used as a routine quality-control technique and also has the potential to reveal otherwise hidden patterns of failure in PK model applications.

## 1 Introduction

The application of pharmacokinetic (PK) modelling techniques to the analysis of dynamic contrast-enhanced (DCE-) MRI data is becoming widespread and can provide quantitative measurements of tumour perfusion and capillary wall permeability [1], [2]. It is however becoming increasingly apparent that none of the most commonly used models fit to DCE-MRI data adequately in all organ and tumour types [3], [4], [5], [6]. Optimal analysis may therefore require testing multiple models in order to decide which gives the best fit to the data. This can be done on a voxel-wise basis to fit appropriate models to differing anatomical sites within the same imaged region.

PK model results are usually presented as pixel-wise parameter maps. Statistical methods such as histogram analysis are commonly used to extract tumour-wide results. Clearly it is essential to differentiate between pixels where the PK model converges satisfactorily from those where convergence errors occur, for the purposes of histogram formation.

Other researchers have approached this problem by constructing maps of goodness-of-fit, expressed as χ^2^, and this is certainly a useful technique [7]. In an extension, Banerji et al. [3] apply the idea of mapping the Akaike information criterion (AIC) score to determine the better of two model-fits across the diseased liver. The current study broadens this concept and introduces a more comprehensive form of error category mapping. It will be shown that this can highlight important additional information in an easily examined pictorial form.

## 2 Methods

### 2.1 Data acquisition

This data analysis formed part of a larger multi-parametric oncology study for which ethical approval from the local research ethics committee had been received. Eleven patients with histologically proven metastatic renal cell carcinoma with clear cell component (mRCC) were recruited, each giving informed written consent. The patients underwent 4 identical MRI examinations on a GE 1.5 T system (Signa HDx, GEHC, Waukesha, WI). Two base-line examinations were performed prior to bevacizumab treatment (visits ‘b1’ & ‘b2’) separated by a time interval of at least 24 hours. (Two further examinations were performed after treatment though these data were not utilised in the analysis reported here.) At each MRI examination, 0.1 mmol/kg of Gd-DOTA (Dotarem, Guerbet, Paris, France) was administered by power injector at a rate of 3 ml/s.

Before the contrast agent injection, T_1_ mapping data were collected with a series of single measurements from a 3D fast spoiled gradient echo sequence.

The dynamic series was obtained using a 3D fast spoiled gradient echo sequence with the following parameters: TR 4.0 ms; TE 1.7 ms; flip angle = 18°; 0.7 NEX; field of view 35–40 cm; acquisition matrix 160 × 160 × 10-14; slice thickness 5 mm; parallel imaging (ASSET) acceleration factor 2; pixel bandwidth = 326 Hz. The sequence had a temporal resolution of approximately 3 seconds and a total acquisition time of 10 minutes (181 images). A double oblique orientation was chosen to include the descending aorta in the volume as well as the tumour of interest.

The T_1_ mapping data were acquired using a protocol with identical parameters to the dynamic series acquisition but without parallel imaging, repeated across multiple flip angles (MFA) [1°, 3°, 5°, 10°, 15° & 20°].

### 2.2 Image analysis

All image analysis was carried out using custom software written in MATLAB (Mathworks, Natick, MA) and C++.

**Fig. 1:**
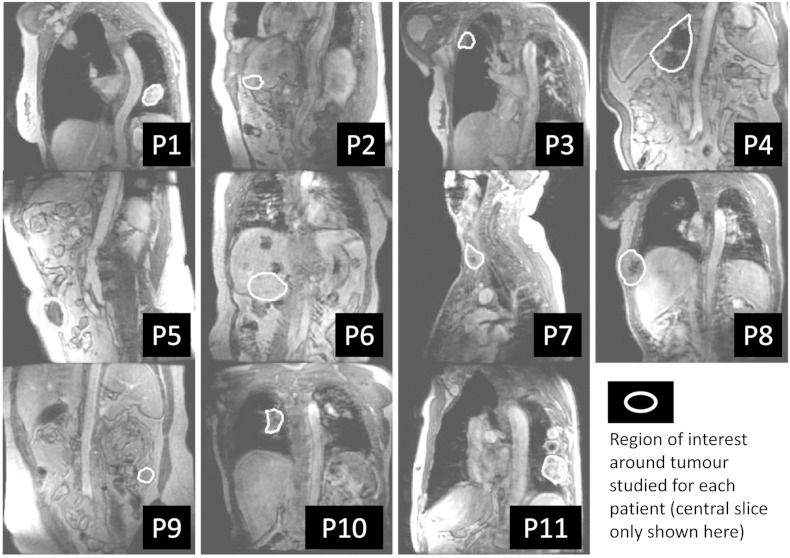
Sample images showing the site of metastatic disease studied for each of the n = 11 patients.

To compensate for the effects of respiratory motion, the dynamic images were aligned using a non-linear registration algorithm using normalised Matte’s mutual information [8], [9]. The MFA images were also registered to the aligned dynamic series. Tumour outlines (see Fig. 1 for examples) were drawn on each slice.

T_1_ maps were calculated from the MFA data by non-linear curve-fitting to the spoiled gradient echo equation [10].

The signal data were extracted on a pixel-by-pixel basis within the ROIs delineating the tumour. This process was repeated across all the image slices containing sections of the tumour. Signal-to-Gd-concentration ([Gd]) conversion was achieved as described in reference [11] using the standard spoiled gradient echo equation [10] and the fact that [Gd] has a linear relationship with the tissue relaxation rate. A value of 3.6 l mmol^− 1^ s^− 1^ was used for the relaxivity of Gd-DOTA [12].

### 2.3 Pharmacokinetic modelling

Using AIFs extracted or derived as detailed below, the ‘extended Tofts model’ (eTM) [13] was fitted to the [Gd] uptake curves to yield parameter maps within the tumour ROIs for *K*^trans^, *v*_e_ and *v*_p_.

The eTM is described by the following equation:

(1) $$C_{t}\left(t\right)=v_{p}.C_{p}\left(t\right)+K^{\mathrm{trans}}.\int_{0}^{t}C_{p}\left(t^{\prime }\right).e^{-\left(K^{\mathrm{trans}}/v_{e}\right)\left(t-t^{\prime }\right)}dt^{\prime }$$

As described in detail in reference [13], *K*^trans^ is the transfer constant (and relates to the perfusion of blood to the tumour and/or its capillary wall permeability), *v*_e_ is the fractional extravascular extracellular volume and *v*_p_ is the fractional plasma volume. *C*_t_(*t*) is the tissue Gd uptake curve and *C*_p_(*t*) the AIF.

Model-fitting employed a trust-region reflective non-linear least squares algorithm [14] implemented by a built-in function in the MATLAB Optimisation Toolbox and was repeated three times, each repetition employing a different AIF form:-

1) a model AIF constructed from blood-sampled data from Fritz-Hansen et al. [15] concatenated with data from Weinmann et al. [16]
2) the AIF measured individually from the dynamic series collected at each patient visit
3) a sample-average AIF constructed from the individual AIFs measured over all 22 relevant patient visits

### 2.4 Error-category maps for pictorial error analysis

In fitting the PK model to each voxel’s data, the following tests were performed to check the reliability of the process. These included, in the following order:-

• A check that the standard deviation of the [Gd] levels in the voxel uptake curve was less than a set limit (in practice this was set at 5 mM). This excluded very noisy data from the fitting process.
• A check that the calculated [Gd] values were real
• A check that the mean [Gd] was greater than a threshold value (in practice the threshold was set to 0.01 mM)
• A check that the non-linear curve fitting process converged satisfactorily
• A check that *v*_e_ ≤ 1
• A check that *v*_p_ ≤ 1
• A check that the parameter values returned were not unreasonably large (in practice a threshold of 10 min^− 1^ was applied to *K*^trans^ and 15 min^− 1^ to *k*_ep_ = *K*^trans^/*v*_e_)

Model convergence was defined in this case as formal convergence of the fitting function or, failing that, the relative step-size or relative change in residuals being less than 10^− 6^. The number of iterations was limited to 100.

A pixel-wise map was constructed showing various conditions of ‘errors in signal-to-[Gd] conversion’ and ‘lack of fit of the model’ in colour codes. These maps are termed ‘error-category maps’.

## 3 Results

The anatomical sites of the metastatic tumour regions are illustrated for all 11 patients in Fig. 1. Most lesions were subject to substantial motion with respiration.

**Fig. 2:**
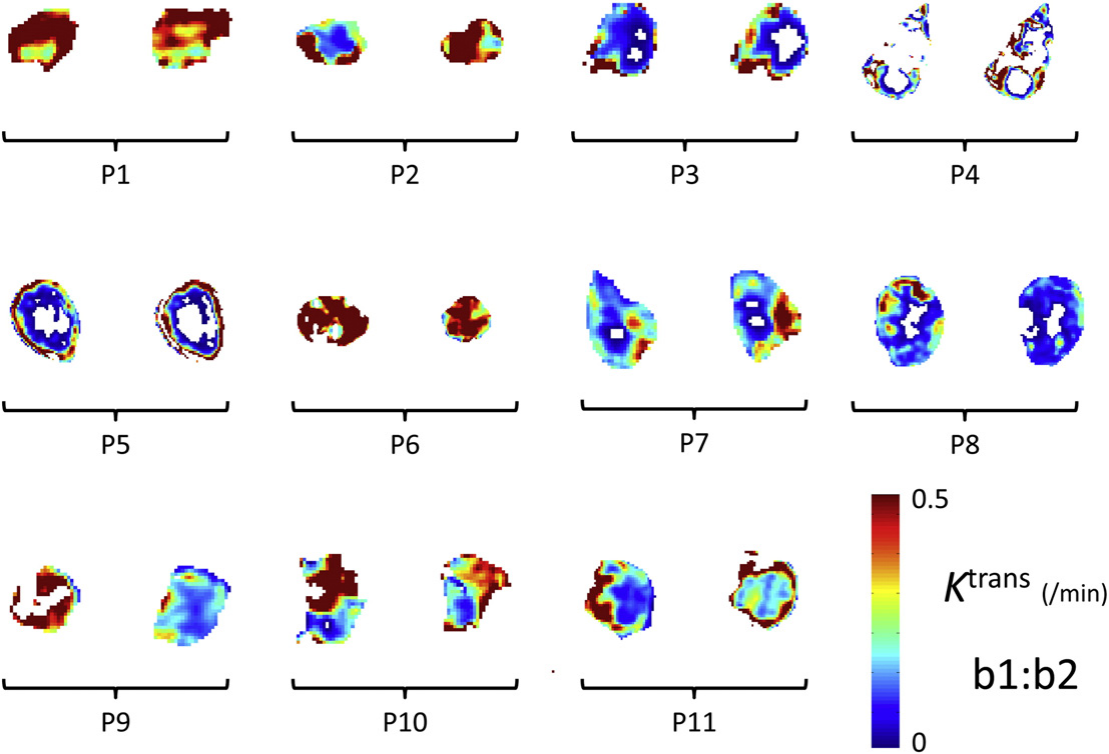
*K*^trans^ maps shown by patient tumour (central slices). Two images with matched locations are shown for each tumour from base-line (pre-treatment) scans ‘b1’ and ‘b2’. White voxels within the peripheral outline correspond to an error-condition in data-analysis (see Fig. 4 for details). (A sample-average AIF was used in the construction of these maps).

Sample *K*^trans^ maps for both pre-treatment scans are shown in Fig. 2, using the sample-average AIF and showing a central slice in each. The maps are broadly similar in pixel-value and heterogeneity between examinations though patients ‘P2’, ‘P9’ and ‘P10’ might be considered exceptions to the rule.

**Fig. 3:**
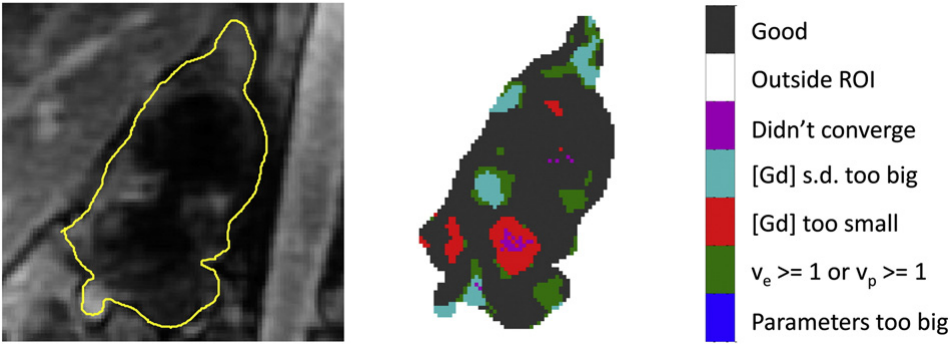
(Left) dynamic series image of a tumour (patient ‘P4’) at full tissue enhancement (ROI outline is shown in yellow); (right) the associated error-category map indicating convergence categories of signal-to-[Gd] conversion and subsequent curve-fitting to the PK model.

Fig. 3 shows a heterogeneous error-category map (taken from a slice through a tumour in patient ‘P4’) together with a fully enhanced image from the dynamic series on which it was based. This tumour image section shows evidence of ‘good’ pixels (black) together with pixels demonstrating non-convergence of the model (magenta), no uptake of [Gd] (red), unreasonably variable [Gd] (cyan) and ‘*v*_e_ ≥ 1 or *v*_p_ ≥ 1’ (green).

**Fig. 4:**
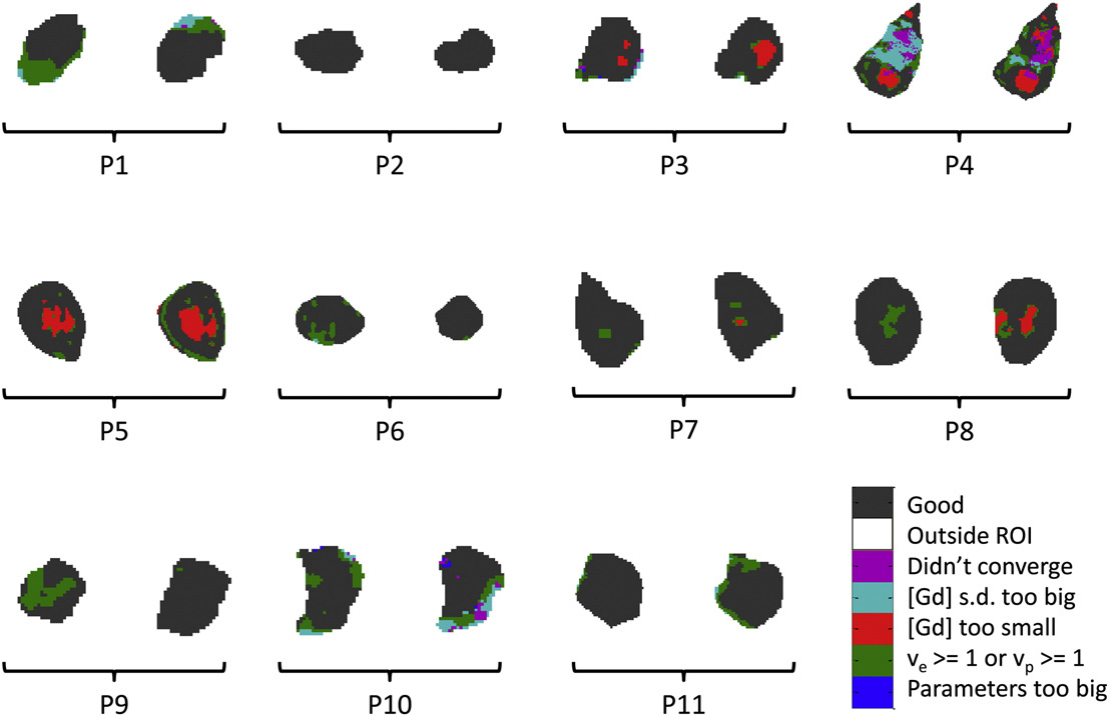
Error category maps for central slice through all 11 patient tumours: pre-treatment scan results compared side by side (b1:b2) for each patient. (A sample-average AIF was used in the construction of these maps).

Sample error-category maps for both pre-treatment scans for all patients are shown in Fig. 4; the maps are taken from an approximately central slice through the tumour in each case and were generated using the sample-average AIF. It can be seen that signals from the majority of pixels gave rise to model convergence (black areas in the maps). There are some regions with no discernible enhancement (red), especially in patients ‘P3’, ‘P4’, ‘P5’ and ‘P8’. The condition ‘*v*_e_ ≥ 1 or *v*_p_ ≥ 1’ (green), is also apparent in many of the tumours.

**Fig. 5:**
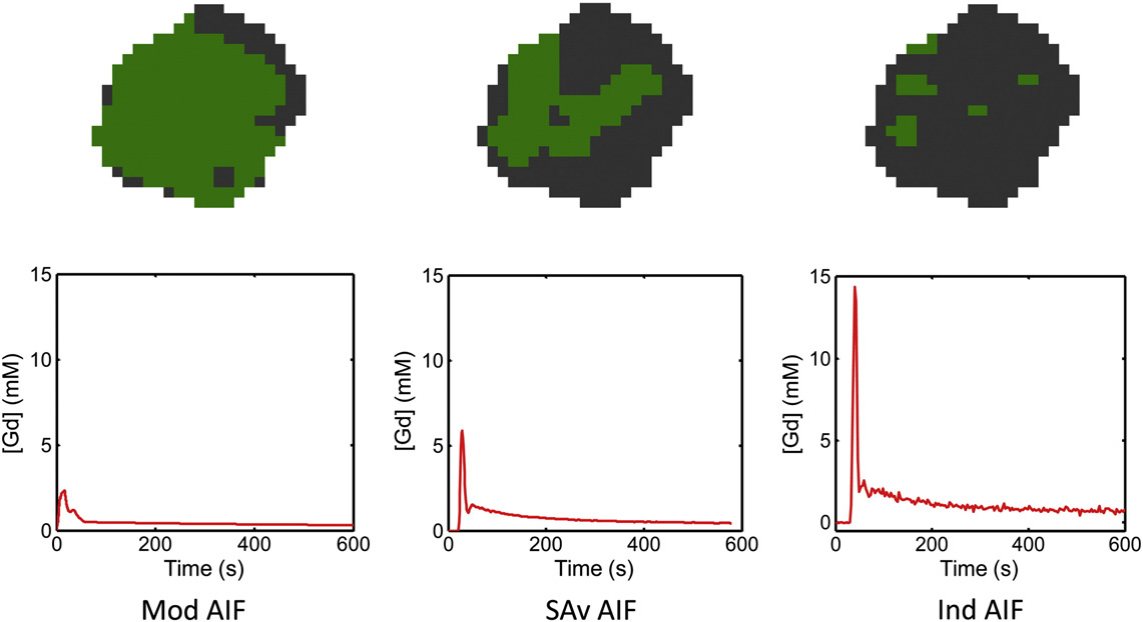
Error-category maps for a central slice through a tumour in patient ‘P9’, examination ‘b1’ (first pre-treatment scan). These are shown for model (Mod), sample-average (SAv) and individually-measured (Ind) AIFs. Green (lighter grey) areas indicate where the model fit gives the un-physical result v_e_ ≥ 1. This error condition decreases in frequency with the relative magnitude of the AIF.

The error-category maps for patient ‘P9’ particularly show evidence of the condition ‘*v*_e_ ≥ 1 or *v*_p_ ≥ 1’ (green areas). Further break-down of error categories revealed that it was the *v*_e_ ≥ 1 error condition which was present (rather than *v*_p_ ≥ 1). Fig. 5 shows the corresponding maps for this patient when analysed using all three types of AIF (model, individual and sample-average). It can be seen that the error condition *v*_e_ ≥ 1.0 is very sensitive to the form of the AIF used.

## 4 Discussion

A method has been described for producing error-category maps in the course of PK analysis of DCE-MRI data. Descriptive examples of the utility of such maps have been given relating to a repeatability study in PK mapping of mRCC tumours.

The error-category maps serve two purposes: firstly, the success of the model in fitting the data can be assessed immediately and secondly, the map can be used as a mask in data extraction from the PK model parameter maps. For example, referring to Fig. 3, the black and red areas refer to ‘convergence of the model’ and ‘no discernible uptake of Gd’ respectively. Forming a mask from these areas combined would allow histogram assessment of ‘valid tumour voxels’ in the PK parameter maps, where no uptake of Gd is regarded as a valid perfusion outcome (i.e. *K*^trans^ = 0).

The error-category maps shown in Fig. 4 are those obtained using the sample-average AIF. This AIF was selected for these illustrations because it gave rise to fewer ‘bad pixels’ (i.e. those coloured other than black on the maps) than either the model AIF or individually measured AIFs. This in itself is a useful result since it indicates in a very direct way that the sample-average AIF is the most reliable form to use in this context.

Subjective examination of Fig. 4 shows that the heterogeneity of the error-category maps is reasonably repeatable between patient visits, though patient ‘P9’ shows the greatest variability. This general trend is interesting since it indicates that more systematic sources of error rather than random measurement errors are dominant in the image analysis process.

It can also be observed from Fig. 4 that the signal-to-[Gd] conversion process and/or the PK model-fitting process failed to execute successfully in some tumour voxels in most patients. The reasons for this, where they can be determined, are varied. They include the presence of genuinely differing physiological states, the influence of the AIF used and acquisition factors such as SNR and tumour motion. It should however be noted that the data from the majority of voxels in each tumour were adequately fitted by the eTM (though the tumour in patient ‘P4’ is a possible exception).

Of the voxels which did not allow good PK model-fitting, those labelled with the condition ‘[Gd] too small’ (red areas on Fig. 4) are straightforward to explain. These regions had low average [Gd] levels resulting from no significant uptake of contrast agent. This was probably due to necrosis in the centre of the tumours concerned. Whilst this hypothesis cannot be proven with this data, it is supported by the fact that the red regions lie at the centre of the tumour ROIs affected.

Patient ‘P4’ provided very noisy data, this probably being due to the combined effects of image registration inaccuracies and a non-uniform tumour. Noisy curves would naturally trigger the ‘[Gd] s.d. too big’ error (cyan areas in the error-category maps) or, failing that, would be expected to cause non-convergence of the model-fitting process (magenta). Note that the ‘[Gd] s.d. too big’ (> 5 mM) condition would not exclude valid curve-fits in these patients since they all had a maximum tumour uptake of < 1 mM of Gd. (A more sophisticated measure could be adopted which compares the variance of the uptake curve after contrast agent administration with the variance before. This relies on a good automated process for determining the bolus arrival time which is not easily determined, especially in noisy data.)

The green areas in the error-category maps were found on further investigation to correspond to the non-physical condition *v*_e_ ≥ 1 (rather than the equally non-physical *v*_p_ ≥ 1). Referring to the eTM equation [13] [see Eq. (1)], if it is assumed that an uptake curve *C_t_*(*t*) fits the model well with a defined AIF, *C_p_*(*t*), we can consider how this data might be fitted using a proportionately smaller AIF. It seems reasonable to assume that, if *C_p_*(*t*) is decreased universally by dividing by a factor β > 1 then the model would be fitted best with new parameter values *v*_p_(*new*) = β.*v*_p_(*old*) and *K*^trans^(*new*) =β.*K*^trans^(*old*). To keep the exponent constant would imply a corresponding increase in *v*_e_. Therefore, it seems possible that a high value of *v*_e_ (in places perhaps meeting or exceeding the physically meaningful limit of 1.0) could be a consequence of assuming an artificially small AIF.

This view is well supported by a comparison of the corresponding error-category map for patient ‘P9’, examination ‘b1’, using the three types of AIF: the model-AIF (‘small peak’), the sample-average (‘medium peak’) and the individually measured (‘large peak’). The results are shown in Fig. 5 and display an increasing number of ‘good’ pixels with increasing AIF peak magnitude.

The choice of error categories and their exact definition need not be fixed to those suggested above and could be tailored to the needs of any particular study. However, the categories chosen here show the utility of this technique in selecting the AIF to use in fitting the data and the spatial extent and heterogeneity of errors typical in DCE-MRI analyses.

## 5 Conclusions

Whilst other researchers [7] have pointed out the utility of mapping ‘goodness of fit’ measures (e.g. χ^2^) in PK modelling of DCE-MRI data, we believe a pictorial representation of the range of errors encountered in signal-to-[Gd] conversion and PK model application yields additional useful information which can easily be overlooked by an entirely numerical approach.
